# Quotas, and Anti‐discrimination Policies Relating to Autism in the EU: Scoping Review and Policy Mapping in Germany, France, Netherlands, United Kingdom, Slovakia, Poland, and Romania

**DOI:** 10.1002/aur.2315

**Published:** 2020-05-22

**Authors:** Danielle Bunt, Robin van Kessel, Rosa A. Hoekstra, Katarzyna Czabanowska, Carol Brayne, Simon Baron‐Cohen, Andres Roman‐Urrestarazu

**Affiliations:** ^1^ Department of International Health, School CAPHRI Care and Public Health Research Institute Faculty of Health Medicine and Life Sciences, Maastricht University Maastricht The Netherlands; ^2^ Department of Psychology Institute of Psychiatry, Psychology & Neuroscience, King's College London London UK; ^3^ Department of Health Policy Management, Faculty of Health Care Institute of Public Health, Jagiellonian University Krakow Poland; ^4^ National Institute of Public Health Warsaw Poland; ^5^ Institute of Public Health University of Cambridge Cambridge UK; ^6^ Autism Research Centre, Department of Psychiatry University of Cambridge Cambridge UK

**Keywords:** autism, employment, anti‐discrimination, policy, EU

## Abstract

The low employment rates of persons with Autism Spectrum Conditions in the European Union (EU) are partly due to discrimination. Member States have taken different approaches to increase the employment rate in the recent decades, including quota and anti‐discrimination legislation, however, the implications for people with autism are unknown. The purpose of this scoping review was to provide a comprehensive overview of the history of these employment policies, from seven EU Member States (Germany, France, the Netherlands, the United Kingdom [prior to exit], Slovakia, Poland, and Romania), exploring the interdependence on international and EU policies, using a path dependency analysis. The results indicate that internationally a shift in focus has taken place in the direction of anti‐discrimination law, though employment quotas remained in place in six out of the seven Member States as a means to address employment of people with disability in combination with the new anti‐discrimination laws.

**Lay summary:**

Discrimination is partially responsible for the low employment of people with autism. Several approaches have been taken in recent years, such as anti‐discrimination laws and setting a mandatory number of people with disabilities that need to be employed. This study finds that, internationally and in the European Union, the focus was initially on the use of quotas and gradually moved to anti‐discrimination, with both being used simultaneously. ***Autism Res** 2020, 13: 1397–1417*. © 2020 The Authors. *Autism Research published by International Society for Autism Research* published by Wiley Periodicals, Inc.

## Introduction

Autism Spectrum Conditions (henceforth, autism) are a heterogeneous group of lifelong neurodevelopmental conditions in which the nature and severity of its characteristics, such as persistent difficulties in social interaction, communication and unusual repetitive patterns of behavior, interests or activities, lie on a continuum [American Psychiatric Association, 2013]. The prevalence of autism is estimated as high as 1–2%, with males being three times more often affected than females [Lai, Lombardo, & Baron‐Cohen, 2014; Loomes, Hull, & Mandy, 2017]. Around 30–45% of persons with autism also have intellectual disability [Lai, Lombardo & Baron‐Cohen, 2014; Baio et al., 2018]. Long term outcomes of people with autism are variable, although the condition is overall associated with a poor outcome in adulthood [Howlin, Goode, Hutton, & Rutter, 2004; Howlin, Moss, Savage, & Rutter, 2013]. In particular, individuals with autism face challenges related to physical and mental health, developing friendships or romantic relationships and gaining and maintaining employment [Billstedt, Gillberg, & Gillberg, 2005, 2007; Eaves & Ho, 2008; Farley et al., 2009; Howlin, et al., 2004]. This results in poor social inclusion and lower quality of life [van Heijst & Geurts, 2015]. Furthermore, different studies affirm a significant increased mortality rate of people with autism in comparison with the general population [Gillberg, Billstedt, Sundh, & Gillberg, 2010; Mouridsen, Brønnum‐Hansen, Rich, & Isager, 2008], as well as an increased risk of dying by suicide [Cassidy et al., 2018; Hirvikoski et al., 2016; Zahid & Upthegrove, 2017]. Thus, autistic people are vulnerable and improving their wellbeing is essential and poses a serious public health challenge.

A well‐known risk factor for adverse health outcomes and poor health is unemployment [Cassidy, et al., 2018; Janlert, Winefield, & Hammarström, 2014; Norström, Virtanen, Hammarström, Gustafsson, & Janlert, 2014; Yur'yev, Värnik, Värnik, Sisask, & Leppik, 2012]. Conversely, it has been reported that paid employment could increase the quality of life of people with autism, since employment has multiple individual and social benefits [Fleming, Fairweather, & Leahy, 2013; García‐Villamisar, Wehman, & Navarro, 2002; Gerhardt & Lainer, 2011]. First, being employed has a beneficial effect on mental and physical health [Ross & Mirowsky, 1995; van der Noordt, IJzelenberg, Droomers, & Proper, 2014]. Second, employment involves integrating into a social network, contributing to society, and being seen as part of a society, therefore increasing an individual's social status [Roux et al., 2013]. Third, paid employment can result in more confidence and independence of autistic people [Roux et al., 2013], and may result in reduced reliance on publicly funded programs, though higher rates of autism employment may require a shift from benefit funding to funding programs supporting people with autism on the work floor [The National Autistic Society, 2016]. Nevertheless, employment remains one of the most desirable achievements for people with autism [Roux et al., 2013; Wilczynski, Trammell, & Clarke, 2013].

Although the beneficial effects of employment on the quality of life of people with autism and for broader society have been known for several years [García‐Villamisar, et al., 2002; Roux et al., 2013], the employment rates of people with autism are still very low in comparison to other disabilities and conditions [Shattuck et al., 2012]. Whereas the total employment rate of people with disabilities in 2011 in the European Union (EU) was 47.3%, Autism‐Europe indicates an overall employment rate for people with autism as low as 10% [Autism‐Europe, 2014]. Surveys completed by people with autism—or people on behalf of the people with autism—indicated employment rates from 32% in the United Kingdom (UK) [The National Autistic Society, 2016] to 45% in the Netherlands [Scheeren, Begeer, Van Wijngaarden, Van Der Jagt, & Mataw, 2019]. Unfortunately, data is not available in every EU Member State. Although paid employment is not possible for every person with autism, a survey from The National Autistic Society showed that 77% of the unemployed participants want to work [The National Autistic Society, 2016]. Identified barriers for employment are partly of the same nature as the characteristics of autism, including difficulties in communication and social interaction, however, discrimination and stigmatization by employers and co‐workers also plays a major role [Autism‐Europe, 2014; Graetz, 2010; Johnson & Joshi, 2016; Lorenz, Frischling, Cuadros, & Heinitz, 2016].

The widely accepted Universal Declaration of Human Rights proclaimed in 1948 that everyone has the right to work and the right for protection against unemployment [United Nations, 1948], making it a human rights problem on top of a public health challenge if people with autism are denied or obstructed (access to) employment. Analyses of special education policy for children with autism by the European Consortium for Autism Researchers in Education [EDUCAUS, 2019] found that the right to education that was incorporated in the UDHR was further elaborated on by other international documents and subsequently implemented in national policy [Roleska et al., 2018; van Kessel, Roman‐Urrestarazu, et al., 2019; van Kessel, Walsh, et al., 2019]. As such, it can be expected that this is also the case for rights surrounding employment—even though this has not yet been comprehensively investigated yet.

EU Member States have indeed taken different measurements in the attempt to assure employment of people with disabilities. The majority of the European countries have some form of an employment quota, with the obligation to employ a minimum percentage of disabled employees within a company or department [Greve, 2009; Waddington, 2000]. In addition, in 1996, Waddington argued that during the time of publication, there was a shift from policies from employment quota policies to anti‐discrimination policies [Waddington, 1996]. Anti‐discrimination law is based on the idea that people with disabilities are equal to persons without disabilities and therefore adopts a more social instead of medical approach of disability [Waddington, 1996]. It prohibits employers to distinguish on the ground of a disability and could therefore be of great value in combatting unemployment of people with autism [Sayce, 2003].

Nearly 25 years later, the question arises if the shift in policies within the EU continued in the same direction as described by Waddington in 1996. Since that publication, 13 countries have joined the EU, and the Charter of Fundamental Rights of the European Union, which guarantees the right for everyone to engage in work in Article 15, has been introduced and given the same legal value as the EU Treaties [European Union, 2000]. Furthermore, international social changes and conventions possibly influenced labor policies in the EU. Research in this area is scarce, especially for the implications of employment policies for people with autism. Previously, Roleska and Roman‐Urrestarazu et al. have identified educational policies within the EU applicable for people with autism and the critical junctures on international and EU level for the development of these policies, however, these are not necessarily identical for employment policies [Roleska et al., 2018]. Analyzing the history of the employment policies within the EU and their value for people with autism can be valuable in understanding the circumstances that have led to the current employment situation and, by extension, to pinpoint areas that should be focused on in future policy developments, such as employment programs for the autism community or programs that educate the workforce on how to address autism [Waddington, Priestley, & Yalcin, 2016].

Therefore, the aim of this study is to map policies pertaining to employment quotas and anti‐discrimination in the EU and investigate the transition from the former to the latter. It also aims to identify the implications of each respective policy for the employment of people with autism specifically. Using a scoping review with a path dependency methodology framework, this study comparatively assesses the quota and anti‐discrimination employment policies on an international, EU, and Member State level from 1944 to 2019.

## Methods

This study used the scoping review method by Arksey & O'Malley to collect data, which provides a rigorous, transparent, and rapid method to comprehensively identify all relevant policies and literature [Arksey & O'Malley, 2005; Levac, Colquhoun, & O'Brien, 2010]. It is particularly suitable for the purpose of this study considering the heterogenous and complex nature of the topic [Arksey & O'Malley, 2005; Pham et al., 2014].

A path dependence method was integrated with the analysis of the scoping review to analyze interdependence of international policy, EU policy, and Member State policy, and to identify critical junctures that might lead to a change in development of employment policies. The theory of path dependence provides an analytic framework to explain and assess constant changing outcomes, such as policies [Antonelli, 2009]. It emphasizes the importance of history of institutions and their interdependence and assumes that decisions from the past still affect those processes of the future [Liebowitz & Margolis, 1995; Pierson, 2000; Schmidt & Spindler, 2002]. This is especially a suitable methodology for public policy within the EU, because of the multi‐level governance structure, which creates many paths which can affect each other [Holzinger & Knill, 2002]. Since in path dependency the necessary conditions for current outcomes occurred in the past, a crucial moment is that of the critical juncture, or triggering event, which started development along a particular path [Pierson, 2000]. It is the aim of path dependency to find these junctures. The combination of a scoping review with a path dependence analysis as the conceptual model for analyzing policies was previously validated by Roleska and Roman‐Urrestarazu et al. [2018].

### *Data Collection*

Seven countries were chosen to provide a sample of different employment policy initiatives across the EU, based on accession date to (a precursor of) the EU, the size of the economy due to its relation with employment and gross domestic product (GDP) *per capita*. These include Germany (accession 1958), France (accession 1958), the Netherlands (accession 1958), the United Kingdom (accession 1973, exit in 2020, after the analysis of this study was completed), Poland (accession 2004), Slovakia (accession 2004), and Romania (accession 2007) [European Parliament, 2018]. Germany, the UK and France have the largest economies among the countries under study, Poland has the largest economy of all the 2004 and 2007 enlargement countries and Romania has a larger economy than Bulgaria, which also joined the EU in 2007 [Eurostat, 2019b]. The Netherlands and Slovakia have a relatively high GDP *per capita* in comparison to countries of the same entrance date [Eurostat, 2019a]. In addition, the United Nations (UN) and the EU were included because of their direct and indirect influence on public policy. The comparative assessment of these international institutions and countries provides the opportunity to explore the role of different institutions and histories in the development of employment policies for people with autism.

To identify all relevant policies, a multi‐level document search was conducted. All databases were searched between May 23 and June 13, 2019. For the UN one legal database was searched (http://www.ohchr.org), as well as for the EU (http://eur-lex.europa.eu), two for France (https://www.legifrance.gouv.fr/ & https://recherche.conseil-constitutionnel.fr), one for Germany (http://www.gesetze-im-internet.de/), the UK (http://www.legislation.gov.uk/), the Netherlands (https://wetten.overheid.nl/), Slovakia (https://www.slov-lex.sk/), Romania (http://legislatie.just.ro/), and three databases for Poland (http://monitorpolski.gov.pl/, http://dziennikustaw.gov.pl/ and http://isap.sejm.gov.pl/). Two searches were conducted per database, using the following terms: (a) “employment AND disability” and (b) “autism” and translated to the official language of the researched national database. Search terms evolved during the search so that lingual differences were accounted for [Arksey & O'Malley, 2005]. A second search strategy was created for electronic databases MEDLINE/PubMed (1946–present) and Web of Science (1988–present), to broadly cover the disciplines of disability and employment. The following search was carried out and tailored according to the database and country, however, search terms were not translated at this stage: “employ* OR work OR job” AND “autism OR ASC OR ASD OR disability OR handicap” AND “policy OR law OR legislation OR legislative” AND [country name]. Finally, Supporting Information was found through an additional reference search of the retrieved documents after the first two stages of data collection. All retrieved documents were managed in Excel (Microsoft). Documents in a language other than English, Dutch or German were translated with the electronic translating service Google Translate.

### *Eligibility Criteria*

An overview of the eligibility criteria is provided in Table I. We chose to not use documents from the German Democratic Republic because, after the unification, this part of Germany used the former policies from the Federal Republic of Germany. As such, the policies by the German Democratic Republic were not considered suitable for path dependency analysis. Also, as this article involves a scoping review, no assessment of quality criteria was conducted [Arksey & O'Malley, 2005].

**Table I:** The Eligibility Criteria Used in This Study

Criteria	Specification
Inclusion	Documents published from 1944 onwards, considered the beginning of the end of the Second World War in Europe
	Governmental documents and scientific articles
	Scope of included documents was limited to policies relating to employment and disability
	National policies had to be legally binding, though international and EU policies did not have this criterium
	Documents from the legal databases and reference search, that were published in one of the European Union's 24 official languages^a^
	Documents from the research databases only when written in English, due to time restraints
	For Germany, documents from the Federal Republic of Germany
Exclusion	Documents from the German Democratic Republic were excluded

a Based on an overview by European Union [2016].

### *Data Analysis*

The data analysis was conducted in accordance with the path dependency framework. All retrieved policy documents were stored and analyzed separately according to the source; UN, EU, and each of the seven Member States. The following step in the analysis contained the dividing into clusters of (a) employment quota, (b) anti‐discrimination policy or (c) other employment‐related legislation. Within these clusters the policies were analyzed by country and per cluster, to enhance analysis for interdependence with other levels or countries. Thereafter, passages concerning employment and disability were critically appraised for effects and implications.

## Results

**Figure 1:**
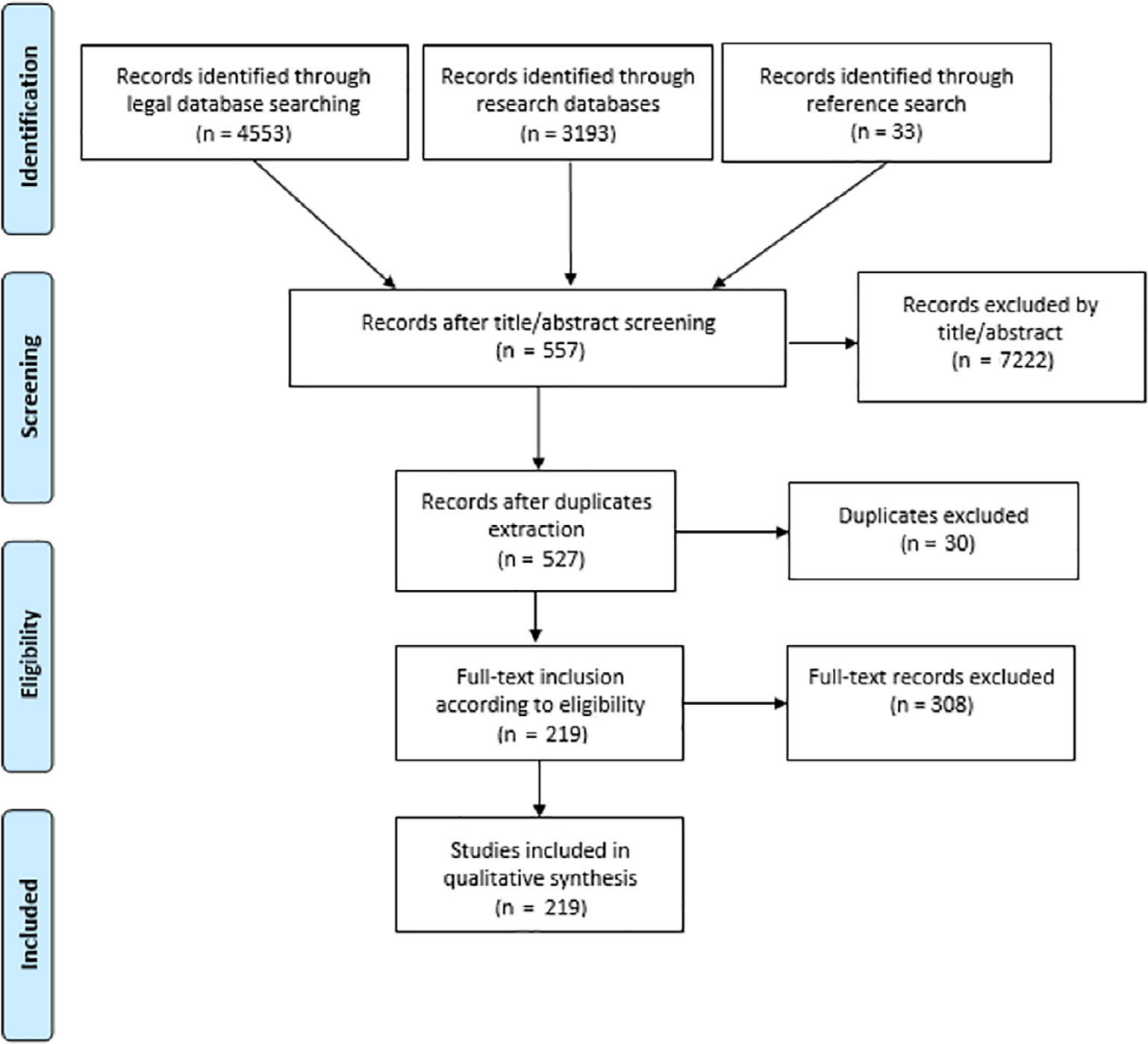
Overview of the data inclusion process. Adapted from the PRISMA group (2009).

Through the initial database searches 4,553 policy documents and 3,193 articles and reports were identified, resulting in a total of 7,746 hits. A reference search of these documents resulted in the inclusion of another 33 documents. After title screening, 557 documents were included, followed by the extraction of 30 duplicates. Through full‐text screening, another 308 documents were excluded, leaving 219 documents included. The inclusion process is presented in an adapted PRISMA Flowchart (Fig. 1).

The following section presents the findings of the yielded policies. First, the international and EU junctures in regard to employment, disability, autism, and (anti‐)discrimination rights are presented (Table II). Second, quota and anti‐discrimination legislation from the retrieved policies are explained per country (Table III). In addition, where applicable these policies were put in the context of other employment‐related legislation such as constitutions.

**Table II:** A Synopsis of Policies Ratified by the United Nations and European Union on the Topic of Employment for People with Disabilities

	Year	Policy	Description
United Nations	1948	Universal Declaration of Human Rights	Considered to be the foundation of human rights and anti‐discriminatory law. Enumerated the fundamental rights of humans, which are stated to be universal, therefore including autistic people. Expressed the right to work and protection against unemployment for everybody, as well as the right for free choice of employment, favorable working conditions and the right for equal pay for equal work.
	1969	Declaration on Social Progress and Development	Emphasized the necessity of the protection of the rights and assurance of the welfare of the physically or mentally disadvantaged people.
	1971	Declaration on the Rights of Mentally Retarded Persons	Established that a person with disability has the same rights as typical people to the maximum degree of feasibility. Expressed the right to perform productive work or to engage in any other meaningful occupation to the fullest possible extent of his capabilities. States a right to protection from exploitation, abuse and degrading treatment.
	1975	Declaration on the Rights of Disabled Persons	Disabled people have the right, according to their capabilities, to secure and retain employment or to engage in a useful, productive and remunerative occupation and to join trade unions. Disabled persons shall be protected against all exploitation, all regulations and all treatment of a discriminatory, abusive or degrading nature.
	1981	International Year of Disabled Persons	Different national assemblies of disabled people came together and found common commitment to fight against the discrimination and towards the rights of disabled people.
	2006	Convention on the Rights of Persons with Disabilities	Agreed on equal rights for people with disabilities and the right of persons with disabilities to work, on an equal basis with others without discrimination on the grounds of a disability. Ratifying countries had to implement legislation according to the protocol of the CRPD, however for employment the scope was fairly limited.
European Union	1971	European Social Fund	In case of long‐term unemployment, assistance would be granted to people with disabilities.
	1981	Framework for the Development of Community Action for the Social Integration of Disabled People	Mentions the employment situation of people with disabilities, though does not establish anything specifically.
	1986	Council Recommendation of 24 July 1986 on the employment of disabled people in the Community	Recommended Member States to adopt policies to:(1) promote the fair opportunity of disabled people in the labor market; (2) combat discrimination in employment; and (3) undertake “positive action” in the form of employment quotas for disabled people.
	1992	Charter for Persons with Autism	Emphasizes the right to meaningful employment without discrimination, with a fair income and access to all assistance and support services necessary to live an independent dignified life. Formally adopted by the European Parliament in 1996.
	1994	White Paper on European Social Policy ‐ A way forward for the Union	Since it could hinder the free movement within the single market, the EU did not have the competence to adopt legislation to combat discrimination. This document, as a response to this lack of competence, contained the advice to consider the possibility of amending the Treaties to provide competence to combat discrimination on the grounds of disability.
	1997	Treaty of Amsterdam	Made it eventually possible for the EU to take appropriate action to combat discrimination based on disability.
	2000	Directive Establishing a General Framework for Equal Treatment in Employment and Occupation (2000/78/EC)	Was established with the purpose of combating discrimination in employment. Member States had the obligation to implement legislation in accordance with its goals. The most important provisions of this Directive for people with autism is the right of reasonable accommodation to enable a person to have equal access to and participate in the workplace and the prohibition of discrimination in employment with the principle of equal treatment. Positive action, as established in 1986, was considered to be allowed when it would ensure full equality in practice.
	2000	Charter of Fundamental Rights of the European Union	Combined previous established fundamental rights, values and freedoms into one legally binding document Several rights are reaffirmed:(1) The right for everyone to work; (2) Equality before the law; and (3) Prohibition of discrimination on the grounds of, amongst others, disability.
	2010	European Disability Strategy 2010–2020	Has its foundations in the CRPD while considering the fundamental rights of EU citizens established in Charter of Fundamental Rights of the EU and the Treaty on the Functioning of the EU. Aims to empower people with disabilities and improve their social and economic situation, so that they can benefit fully from participating in society. The EU recognizes the poor employment rate of people with disabilities and claims it will act in supporting national efforts to help integration in the labor market using the European Social Fund.
	2015	Written Declaration on Autism	Particularly focused on education and employmentRecognizing the poor employment status, the European Parliament calls upon the Commission and Council to act on promotion of employment opportunities for people with autism.

*Note*. EU stands for European Union; CRPD stands for Convention on the Rights of Persons with Disabilities.

**Table III:** A Synopsis of Each Respective Policy of All Countries Under Study with a Distinction Between Policies Focused on Quotas and Anti‐discrimination

		Year	Policy	Description
Germany	Quota	1953	Severely Damaged Act	Focused on injured veterans and victims of the National Socialist Regime. Every employer with a minimum of seven employees had to provide 8 or 10% of their workplaces to disabled or “damaged” persons, according to their sector. All employers within the scope of the Act who did not meet the quota were obligated to pay a monthly levy for every unfilled quota place, not releasing them from their obligation, from which the proceeds were put toward supported employment services for disabled people
		1974	Severely Handicapped Persons Act	Replaced the Severely Damaged Act. Employers from both the public and private sector with 16 or more employees were obligated to provide a minimum of 6% of the workplaces to severely disabled people. Noteworthy was the change in definition of disability, to fall under the scope of the Act a person could now be physically as well as mentally disabled but had to have a minimum of 50% reduction of the working capacity. Individuals who were particularly difficult to employ or disabled persons who received vocational training within the company could be counted as occupying more than one quota workplace. The levy system remained similar to the Severely Damaged Act.
		2001	Book IX of the Social Code	Combined German rehabilitation and disability law. Maintained the quota system. The definition of disability was expanded to include more types of disabilities, including different degrees of disabilities.
	Anti‐discrimination	1994	Amendment of the Constitution	Now prohibited disadvantaging on the grounds of a disability. Unfortunately, this change had little impact, since it could not initiate a new anti‐discrimination law unless it could be demonstrated that disabled people would continue to be disadvantaged.
		2001	Book IX of the Social Code	Provided anti‐discrimination legislation, though this was only applicable for discrimination by employers against severely disabled people.
		2002	Disability Equality Act	A response to the 2000/78/EC Directive. Purpose was to eliminate the disadvantages that people with disabilities face in life and promote their equal participation in society, to enable a self‐determined lifestyle. Includes provisions regarding the ban on discrimination of disabled people by public authorities and the obligations for barrier‐free buildings of the federal government and public transport, but did not address discrimination in the employment situation
		2006	General Equal Treatment Act	Addressed discrimination in employment directly as well, with the provision that employees cannot be disadvantaged because of race, ethnic origin, sex, religion, age, sexual identity, or disability. To assist people who feel disadvantaged based on the previously mentioned reasons, the Act introduced a Federal Anti‐Discrimination Agency. Was the first anti‐discrimination legislation for all people with disabilities in employment and the final provision based on the implementation of Directive 2000/78/EC into German national law.
France	Quota	1957	Act for the Employment of Disabled People	Replaced a previous quota established after the First World War. Whereas the former act coverage was limited to disabled veterans, widows and orphans, the new Act covered all those recognized as “handicapped” by a Commission, which was previously established. Describes the prioritizing of employment of people with mental or physical disabilities up to a certain percentage by Order of the Minister of Labour and Social Security, in such a way that this percentage ensures the right to work of all disabled persons. A set percentage was not mentioned in the Act but differentiated over the years between 3 and 6%.^a^Did not have the desired effects on the employment of people with disabilities, probably due to poor enforcement.^a^
		1975	Disability Orientation Act of June 30 1975	For the first time sought to address all the needs of people with disabilities, from education to employment and housing, to accessibility of public buildings. Imposed an obligation of integrated employment. However, it did not set up a viable quota for employment of people with disabilities.
		1987	Act for the Employment of Disabled Workers	Amended the earlier established French Labour Code (1973). This amendment introduced a new quota, obligating both public and private employers with a minimum of 20 workers to provide at least 6% of the workplaces to people with disabilities or pay a levy. Employers had to conform to the quota within 3 years after enforcement, increasing from 3% in 1988 to 4% in 1989 and 6% in 1991.^a^
		2005	Act of Equal Rights and Opportunities, Participation and Citizenship of Persons with Disabilities	Amending the French Labour Code, reconfirmed the quota of 6% for companies with at least 20 employees. The levy for companies that does not meet this quota was raised significantly in order to increase the employment rate of people with disabilities. The income generated from this levy presently still goes toward a fund for the professional integration of people with disabilities.
	Anti‐Discrimination	1958	Constitution of the French Republic	Stated that all citizens are equal for the law, without distinction of origin, race, or religion, though disability is not mentioned.
		1990	Act on the Protection of Persons against Discrimination on Ground of their State of Health or Disability	Provided an amendment the Penal Code in such a way that it became a criminal offense to discriminate against people based on state of health or disability. It was considered forbidden for an employer to refuse employment or dismiss an individual based on a disability, unless the refusal of hiring is a result of medical unfitness of the person according to the Labour Code of 1973. It was considered a criminal offense for providers of goods and services to refuse to supply to a disabled person on the ground of their disability.
		2005	Act om Equal Rights and Opportunities, Participation and Citizenship of Persons with Disabilities	Contains the necessary legislation to implement the 2000/78/EC directive, such as the provisions of appropriate adjustments for a disabled employee to carry out a job corresponding to their qualifications.
Netherlands	Quota	1947	Act of Placement of Disabled Workers	Employers from both the public and private sector with more than 20 employees were required to employ a set quota of 2% disabled workers. A person with a disability, or “less‐able bodied”, was defined as someone who, as a result of mental or physical impairments, restricted is in the ability to sustain themselves through employment. Injured war veterans where specifically mentioned as people who would fall under the Act. A person with a disability could choose to register as such, to be placed within the protection of the Act. In 1981, the Dutch House of Representatives concluded that this Act was difficult to enforce, due to an inadequate definition of “less able‐bodied.”Replaced by the Handicapped Workers Employment Act in 1986.
		1986	Handicapped Workers Employment Act	Extended coverage to all people receiving invalidity pensions or disability benefits.Introduced a new form of voluntary quota, between 3 and 5% to be achieved over the following 3 years. Stated that the voluntary scheme would be replaced after 3 years by a legislative obligation, enforced with a fine, if the quota was not reached.
		2014	Amendment to the Social Insurance Financing Act in connection with a levy in the event of non‐compliance with the quota objective	Illustrates that, when the employment rate of disabled people showed little improvement by 1989, no obligatory quota was introduced. The government rather concluded that such a quota was not a feasible policy in practice and continued the quota on a voluntary basis until 2005, when the inactive quota system was abandoned.
		2015	Jobs and Jobs Quota (Work Disabled Persons)	Reintroduced the quota system for people with disabilities who fall within the Participation Act, to increase the inclusion of persons with disabilities in the Dutch labor market. From 2015 until 2026 a total of 125.000 workplaces should be created for disabled people, 100.000 in the private sector and 25.000 in the public sector, divided over the years. If a sector does not meet the target for the year, a quota is calculated (1,93% for the public sector in 2018) for the minimum of disabled people in their workforce. Employers with more than 45 employers who do not meet the quota that year will be charged a fine.
	Anti‐Discrimination	1983	Dutch Constitution	Contains the provisions that every person in the Netherlands should be equally treated in equal circumstances, discrimination on the grounds of religion, political opinion, race, sex or on any other grounds was prohibited. It is considered a traditional fundamental right with the purpose of protection of the citizens against the government. Thus, under this article, *public institutions* are not allowed to discriminate people with disabilities.
		2003	Equal Treatment (Disability & Illness) Act	Refers to Directive 2000/78/EC and the Dutch Constitution. Disability is not further defined than having a handicap or chronic disease. Prohibits a direct or indirect distinction between persons with and without disabilities, which is elaborated on in relation to employment. Employers are also responsible for the provision of access for people with disabilities, unless this requires unreasonable efforts, and failing to do so is also considered as discrimination. However, positive discrimination is allowed when a regulation or practice has the goal of eliminating or reducing disadvantages related to disabilities and the distinction is proportionate for that purpose.
		2016	Amendment to the Equal Treatment (Disability & Illness) Act	Now in line with the provisions set out in the CRPDNo changes for people with autism were made.
United Kingdom	Quota	1944	Disabled Persons Employment Act	Required all employers from the private sector with 20 or more employees to have at least 3% of their workforce to be registered as disabled, preferably disabled military men and woman. Provided the first definition of a disabled person, including injury, mental or physical disease and congenital deformity, by which the person would be substantially handicapped in obtaining or keeping employment, rather than an impairment specific definition. It was not an offense in itself for an employer to have less than 3% disabled workers, however the employers could in that case only allocate vacancies to disabled people, until the quota had been met. Non‐adherence to the Act could lead to a high fine or up to 3 months of imprisonment.
		1995	Disability Discrimination Act	Abolished the quota system.
	Anti‐Discrimination	1995	Disability Discrimination Act	Made it unlawful to discriminate against people with disabilities in different domains, such as employment and the provision of services. A person with a disability was defined as someone who has a physical or mental impairment, with a long‐term adverse effect on the ability to carry out normal day‐to‐day activities. Changed the focus of employment of persons with disabilities from state regulation to voluntarism and the elimination of barriers for employment. An employer is legally not allowed to treat a person less favorably for a reason related to a person's disability, than he treats a person in without a disability and where he is unable to justify that treatment. However, an employer is allowed to treat a person with a disability more favorably. An employer has, in addition, the duty to make arrangements to minimize physical barriers for disabled people when these barriers lead to substantial disadvantages in comparison with people without disabilities. This Act was originally not applicable for businesses with fewer than 20 employees, but this was withdrawn in an amendment in 2003. No central body was installed for monitoring and enforcement of the Act, although the Act did provide a National Disability Council to advise the Secretary of State on the operation of the ActCan only be enforced by disadvantaged individuals *via* industrial tribunals.
		2005	Disability Discrimination Act	Provided minor changes to adhere to the Directive 2000/78/EC, as well as the duty of public authorities to take a more proactive role in the promotion of equality and inclusion of disabled people, also known as the Disability Equality Duty.
		2010	Equality Act	Consolidated, updated and supplemented the prior Acts and Regulations, that formed the basis of anti‐discrimination law in the United Kingdom. The disability definition remained the similar, containing the need for long‐term negative effects to fall within the scope of the definition of disabled person. Whereas the Disability Discrimination Act contained separate provisions on reasonable adjustments in each of the different parts, the Equality Act sets out the core of this duty with additional context‐specific detail, such as the provision of auxiliary aids. A failure to make reasonable adjustments for disabled people in the workplace is to be considered a form of unlawful discrimination.
Poland	Quota	1967	Ordinance of the Council of Ministers on the Planned Employment of Disabled Persons	Companies were obligated by the Act to employ people with disabilities when an average work efficiency could be attained. Employers in companies with more than 500 employees had to set up rehabilitation committees for disabled workers.
		1991	Polish Act on Employment and Vocational Rehabilitation of the Disabled	Replaced the Ordinance of the Council of Ministers on the Planned Employment of Disabled Persons. Regulated the duties and rights of employers related to the employment of people with disabilities. Established a Minister for Disabled Persons and set up the State Fund for Rehabilitation of Disabled Persons.Disabled persons were defined as persons with a significant degree of physical and psychic or mental impairment, limiting their capacity to work. Employers with a minimum of 50 employees had an obligation to have at least 6% of their employees as having a recognized disability with monthly payments per missing employee, from which the proceeds would go to the State Rehabilitation Fund for the Disabled. In case of a particular difficult to employ person, this person could fill up more than one place after approval by the Ministry of Labour and Social Policy. Another way to meet the quota was to buy products produced by disabled people in sheltered workshops. Financial resources of the Fund were allocated toward rehabilitation of people with disabilities.
		1997	Act on Employment and Vocational Rehabilitation of Disabled Persons	Replaced the Polish Act on Employment and Vocational Rehabilitation of the Disabled. The minimum number of employees for an employer to fall under the quota was reduced to 25. The 6% target remained the same, as did the provision of the State Fund, now named the Polish Fund for Rehabilitation of Disabled Persons.
	Anti‐Discrimination	1952	Constitution of the Polish People Republic	Work is the right, the duty and a matter of honor for every citizen.
		1996	Polish Labour Code	Any discrimination in employment relations on the basis of different ground such as disability, sex and race was unacceptable.
		1997	Polish Constitution	Replaced the temporary constitution that was in place after the fall of the Soviet regime. All persons are equal for the law and have the right to equal treatment by public authorities. Discrimination based on political, social or economic grounds is prohibited. Mentions the freedom to choose employment. Specific criteria for prohibited forms of discrimination are not specified and thus, anti‐discrimination measures especially for people with disabilities are not mentioned.
		1997	Charter of Persons with Disabilities	Recognized as well that people with disabilities have the right to lead an independent life and that they may not be discriminated. People with disabilities have the right to work in an open labor market with conditions adapted to their needs. This Charter was not binding, since it was approved as a resolution and served as a declaration of values, however, all legislation adopted after the Charter could not contradict its terms.
		2004	Amendment to the Polish Labour Code	Changes to implement Directive 2000/78/EC in order to be eligible to access the EU. However, no crucial changes were made.
		2010	Act on the Implementation of Certain European Union Regulations in the Field of Equal Treatment	Made the final provisions of implementing EU directives in this field. Next to anti‐discrimination legislation, it also obliged employers to provide necessary reasonable adjustments to the workplace for disabled persons, in order to increase the equal access and opportunity. Together with the Polish Labour Code, this document provides broad protection from discrimination in the employment field, but very little outside of this field.
Slovakia	Quota	1990	Act on Employment	Defines citizens with reduced working capacity as persons under 65 years of age who had a significant reduced chance of employment due to an unfavorable state of health. Severely disabled people are mainly to be placed in facilities of the unions of the disabled, or in sheltered workshops set up by employers, for which they can receive funding. Employers were obligated to employ disabled people, with a quota for employers of 6%, but no consequences were specified if an employer disregarded the quota.
		1996	Act on Employment	Introduced the requirement for every employer with 25 or more employees to employ people with reduced working capacities. The quota was set on 4%, of which 0,5% had to be people with severe disabilities. Defines citizens with a reduced working capacity as citizens that are either recognized as partially disabled, or who have a long‐term adverse health condition with a limited chance of continuing vocational education. A severe disabled citizen is someone who is only able to work under extraordinary circumstances. It is not stated, however, which organizing body can recognize a person as having a disability. Employers that did not oblige with the quota had to pay a yearly levy for every unfilled quota workplace. The funds retrieved by these levies were paid to the National Labour Office and used as a resource to support the employment of citizens with a reduced working capacity.
		2004	Act on Employment Services	Established a new quota, which is still in force today. Under the new quota, employers with more than 20 employees are obliged to provide 3.2% of their workplaces for persons with disabilities. A different way to adhere to the quota is by contracting companies that employs disabled employees or by contracting persons with disabilities who are self‐employment, or by buying products or services of sheltered workshops. Employers who do not meet the legal requirements must pay a fine of up to 0.9 times the total labor cost of the national average wage, for each vacancy for which a disabled person should have been hired.
	Anti‐Discrimination	1965	Labour Code	Established the right and obligation to work for every citizen, according to his or hers abilities.
		1991	Amendment of the Labour Code	Re‐introduced not only the right to work but also added the free choice of employment and protection against unemployment. Every citizen was entitled to these rights, without discrimination based on race, gender, social origin, age, or, for example, political status was prohibited. Discrimination based on disability was not specifically mentioned.
		1992	Slovak Constitution	Fundamental human rights were guaranteed for all citizens. Provides the general right to fair and satisfactory working conditions and protection against discrimination in employment. Specified the right for special working conditions.
		2001	Amendment of the Labour Code	Discrimination in employment on the grounds of disability became specifically prohibited. Set out the obligation of employers to create working conditions for employees with disabilities in such a way, that they can carry out work achieving the same results as other.
		2004	Act on Employment Services	Reassured the right of access to employment for every citizen without restrictions, direct and indirect discrimination based on, amongst others, disability.
		2004	Anti‐Discrimination Act	Defined discrimination and lays out the same provisions for anti‐discrimination in employment. Stated when a different treatment based on disability in allowed, for example, when a certain job requires a certain health status.
Romania	Quota	1999	Emergency Ordinance on the Special Protection and Employment of Persons with Disabilities	Disabled persons were defined within this Ordinance as persons who, due to physical or mental problems have a disadvantage, which restrict or prevent normal access to social life and who require special protection measure to improve social integration. Sets a quota for public and private companies with at least 100 employees to have a minimum of 4% of their workplaces available for people with disabilities. The workplace should be adjusted according to the needs of the disabled employees. A monthly levy of the minimum wage multiplied by shortage in number of workplaces occupied by disabled persons had to be paid by employers who did not oblige to the quota to the Solidarity Social Fund for Disabled Persons.
		2004	Amendment to the Emergency Ordinance on the Special Protection and Employment of Persons with Disabilities	This minimum employees per company in the private sector to fall within the Scope of the Ordinance was in 2004 reduced to 75, while for the public institutions a minimum amount of 25 was set.
		2006	Act on the Protection and Promotion of the Rights of Persons with Disabilities	Definition of a disabled person and the percentage of obligated work places for people with disabilities was unaltered. The required minimum number of employees to fall within the scope of the quote was set on 50, for public and private institutions. The fine for noncompliance remained similar. Though it was now also possible to fulfill the quota if a company bought products from protective employment units, or sheltered workplaces.
		2017	Amendment to the Act on the Protection and Promotion of the Rights of Persons with Disabilities	Repealed the alternative way for companies to meet the quota. Thus, companies could either comply to the quota or pay a fine.
	Anti‐Discrimination	1964	Romanian Constitution	In force from 1966 until 1991. Had at first no provisions concerning rights of employment or disabilities.
		1971	Act on Employment and Promotion of Personnel from State Socialist Units	The right to work for every citizen was expressed, which in fact was an obligation as well as a right.
		1976	Law concerning the employment of workers capable of working	People who refused to work, were obligated to work in through the state appointed places.
		1986	Amendment of the Constitution	Introduced equal rights for citizens, and prohibited distinction based on nationality, race, sex or religion.
		1991	Constitution of the Romanian Republic	A renewed constitution that was implemented after the revolution. Established the equality of citizens before the law and public authorities as well as the right to work, with a free choice of occupation.
		2003	Amendment of the Constitution	Now also specifically protected people with disabilities. Ensured special protection for people with disabilities by the promise of creating national equality policies.
		2006	Act on the Protection and Promotion of Rights of Persons with Disabilities	Based on the principles of preventing discrimination and equal treatment in the employment field. A person with a disability, or “handicap” under Romanian legislation was defined as a person who could not carry out daily activities in a normal way and who need required support measures, due to a physical, mental or sensory impairment. Despite of this definition, a person with a handicap can only enjoy the rights specified in the law based on official recognition through a handicap certificate. Ensured the right to employment of people with disabilities, any person with a disability that wants to participate in the open labor market is free to do so. Reasonable accommodation has to be provided to all persons with disabilities seeking employment or who are in employment to facilitate the exercise of the right to work, in line with Directive 2000/78/EC. Complements the Romanian Labour Code (adopted in 2003), that lays down other legislation concerning employment. The prohibition of discrimination during the hiring process on ground of disability is not specifically mentioned in this Act.

a Based on a report by Besner, 1994. CRPD stands for Convention on the Rights of Persons with Disabilities.

### *United Nations and European Union Policy*

Several documents have been adopted by the UN that establishes the rights for people with disabilities in terms of their employment and protection from discrimination [United Nations, 1948, 1969, 1971, 1975, 1981, 2006a; Hurst, 2003]. Additionally, with the development of the EU, a baseline of rights for people with disabilities has been established [Autism‐Europe, 1992; Council of the European Union, 1971, 1986, 2000; European Commission 1981, 1994; European Parliament, 2015; European Union 1997, 2000, 2010].

### *Germany*

Germany has adopted several laws that establish quotas for people with disabilities to be employed [Federal Republic of Germany, 1953, 1974, 2001], as well as laws that are focused on anti‐discrimination [Federal Republic of Germany, 1994, 2001, 2002, 2006; Köbsell, 2006]. Notable is that there are no autism‐specific laws. Instead, all identified laws pertain to disability in general.

### *France*

Laws on quotas [French Republic, 1957, 1973, 1975, 1987, 2005; Besner, 1994], as well as anti‐discriminination [French Republic, 1958, 1973, 1990, 2005] for people with disabilities have been passed in France.

Noteworthy is that, in 1987, the mean rate of employment was already at 6% in France and, therefore, the 1987 Act for Employment of Disabled Workers did not have the intention to increase the employment rate of people with disabilities but rather functioned as a measure to standardize employment of disabled individuals [Besner, 1994]. Another way of meeting the quota was possible by indirect employment of disabled workers, such as in the form of a partnership contract with institutions that provided sheltered employment of disabled workers. Additionally, no autism‐specific legislation was identified, only legislation that focuses on the employment of people with disability in general.

### *Netherlands*

Over time, the Netherlands have adopted, abandoned, and readopted numerous policies on quotas [The Netherlands, 1947, 1981, 1986, 2014, 2015], as well as measures to prevent discrimination [The Netherlands, 1983, 2003, 2016].

Although the private sector created a sufficient number of workplaces for disabled persons, the public sector did not in the recent years [The Netherlands, 2018]. However, until now, no fine has been charged and a proposal for pausing the quota levy until 2022 is now in process [The Netherlands, 2019]. Like Germany and France, the Netherlands also only adopted general disability policy in the area of employment.

### *United Kingdom*

Initially, the UK adopted several policies that were aimed at quotas [United Kingdom of Great Britain and Northern Ireland, 1944, 1995]. However, after 1995, this system was abandoned and the focus was put on anti‐discrimination [United Kingdom of Great Britain and Northern Ireland, 1995, 2003, 2005, 2010].

The Disabled Persons Employment Act was in line with the more controlling approach of the British government, which it adopted since the beginning of the Second World War, over certain domains such as employment that had previously been prerogative of the market [Woodhams & Corby, 2007]. Between 1949 and 1975 only six prosecutions took place based on this Act, from which five convictions received insignificant fines [Sargeant, Radevich‐Katsaroumpa, & Innesti, 2018]. Employers did not oblige to the Act due to lack of prosecutions and unawareness of the Act, which led to the feeling of fruitlessness of registration among people with disabilities. This resulted in insufficient amount of registrations, which ultimately led to an impossible situation for employers to oblige to the Act [Sargeant, et al., 2018]. Nevertheless, all these policies remain general disability policies—none of them specifically targeting autism.

### *Poland*

Poland has adopted various policies that were aimed at quotas [Republic of Poland, 1967, 1991, 1997c] and anti‐discrimination [Republic of Poland, 1952, 1997a, 1997b, 1996, 2004, 2010].

No set percentage was established in the 1967 Ordinance for the intake of those disabled workers. A large proportion of disabled people were in fact employed in ordinary establishments until the late 1980s, although these were persons who could obtain a high working capacity [Thornton, 1998]. Employers tried to avoid the more severely disabled, resulting in isolation of the severe handicapped people. As a result of the social and economic changes around 1990 in Poland and other Eastern European countries, from a socialist to a free‐market economy, many ordinary companies collapsed [Thornton, 1998]. A majority of disabled workers lost their jobs and overall employment rates decreased. Consequently, the whole legal and formal system on which the vocational training, social rehabilitation, and employment of people with disabilities ceased to exist [Thornton, 1998]. As with the other countries though, Polish policy does not specifically target autism, but only disability in general.

### *Slovakia*

Former Czechoslovakia and Slovakia both implemented policies aimed at quotas [Czechoslovak Socialist Republic, 1990; Slovak Republic, 1996, 2004b] and anti‐discrimination [Czechoslovak Socialist Republic, 1965, 1991; Slovak Republic 1992, 2001, 2004a, 2004b]. Regardless, these policies targeted disability as a whole, rather than autism specifically.

### *Romania*

Romania, like the other countries under study, has adopted policies on quotas [Romania, 1999, 2004, 2006, 2017] and anti‐discrimination [Romania, 1964, 1971, 1976, 1991, 2003a, 2003b, 2006].

The Act on the Protection and Promotion of the Rights of Persons with Disabilities does not mention if the additional income from the fines from the companies who previously complied with the quota by buying products from sheltered workshops are redistributed toward these sheltered workshops. In practice, companies are more likely to pay the fine than to employ people with disabilities [Bungău, Ţiţ, Popa, Sabău, & Cioca, 2019]. Overall, Romania—like the other countries in this study—only targets disability in general and does not specify autism whatsoever in their policies.

## Discussion

The Second World War can be considered the first critical juncture for modern European employment policies since it led to quota regulation in many countries, as well as inducing the creation of the Universal Declaration of Human Rights. The War left many soldiers disabled and destructed the economy. In the efforts of rebuilding the economy, many governments saw it as their duty to provide for the disabled veterans who lost part of their working capacity while fighting for their country. Germany, France, the Netherlands, and the UK all introduced quota employment policies for people with disabilities in the years after the War, either exclusively or with a preference for war victims [Federal Republic of Germany, 1953; French Republic, 1957; The Netherlands, 1947; United Kingdom of Great Britain and Northern Ireland, 1944]. Subsequently, because an environment of full employment—a situation where there is little‐to‐no involuntary unemployment [Beveridge, 1944]—was retained and considered a desirable situation [Lindbeck, 1997], the scope of quotas developed to include all people with disabilities, including autism.

In contrast, the end of the Second World War did not have the same effect on Poland, Slovakia and Romania (the Eastern States) in which there was no free market economy during that time, and where the citizens had an obligation to work. Quotas were established in all three included countries in the 1990s, in which the Communist regime of these countries ended and the economy changed to a free market (Republic of Poland, 1991; Romania, 1999; Slovak Republic, 1996). Many people lost employment in the transition period, especially people with disabilities and as a response, the governments established quota systems. Therefore, the end of communism can be seen as a critical juncture for employment policies in these countries.

The Universal Declaration of Human Rights was created in the aftermath of the War as well, introducing the universal right to work and protection against unemployment, without distinction based on any ground [United Nations, 1948]. This Declaration can be seen as a critical point in time for employment policies for people with autism, in line with the findings of Roleska and Roman‐Urrestarazu et al. [2018] for its crucial role in the development of education policies for people with autism. It laid the foundation of modern anti‐discrimination law and its influence can be identified in all analyzed Constitutions, as well as in the legally binding Charter of Fundamental Rights of the European Union [2000].

Although internationally in the 1970s and 1980s gradually more awareness was created for the (violation of) rights of people with disabilities, with the Declaration on the Rights of Mentally Retarded Persons, Declaration on the Rights of Disabled Persons, and International Year of the Disabled and France adopting a more holistic approach with the 1975 Disability Orientation Act, no anti‐discrimination legislation was provided specific for people with disabilities in employment until 1990. By the French Act on the Protection of Persons against Discrimination, it became a criminal offense to discriminate against persons based on their disability in employment [French Republic, 1990]. This was followed by a cascade of changes, including the amendment of the German Constitution, prohibiting disadvantaging people on the ground of their disability in 1994 [Federal Republic of Germany, 1994], the Disability Discrimination Act in the UK in 1995 [United Kingdom of Great Britain and Northern Ireland, 1995] and in finally the Polish Labour Code amendment of 1996 [Republic of Poland, 1996], all prohibiting distinction based on disability. With the UK Disability Discrimination Act the previous quota system was abandoned.

Following these changes within the EU, and realizing that a difference in discrimination policies in employment could hinder the free movement within the single market, the EU amended its Treaty in 1997, gaining competence in the field of combatting discrimination [European Union, 1997]. Consequently, the Commission introduced Directive 2000/78/EC [Council of the European Union, 2000], which established a framework for equal treatment in employment and occupation, resembling the UK Disability Discrimination Act and readdressing the fundamental right of the Universal Declaration of Human Rights, leading to (amendment of) comprehensive anti‐discrimination legislation for people with disabilities in employment in every Member State. For Poland, Slovakia and Romania this Directive was implemented around the time of accession to the EU and therefore, membership of the EU and year of accession year were of great importance for the national employment legislation in relation to disabilities. Given the above, the ongoing harmonization of anti‐discrimination legislation can be traced back to the gain of competence of the 1997 Amsterdam Treaty, and therefore, this is considered another critical juncture which influenced employment policy legislation protecting people with autism.

Although the Convention on the Rights of Persons with Disabilities is undisputed important for the improvement of the wellbeing of people with disabilities, it had little impact on the employment policies for EU Member State because of the already implemented provisions of the 2000/78/EC Directive. Therefore, only minor adjustments had to be made to ratify the Convention on the Rights of Persons with Disabilities in the area of employment [United Nations, 2006b].

The abolishment of the quota system by the UK in 1995 and the Netherlands in 2005, following the introduction of anti‐discrimination law, gave the impression that the European focus on anti‐discrimination legislation would eventually replace the quota systems altogether. However, this turned out not to be the case. The Eastern countries all created quotas in the 1990s, resembling the German levy system, and these, as well as the German and French quota systems are still enforced. In fact, the Netherlands recently reintroduced a quota in employment for people with disabilities [The Netherlands, 2015]. Moreover, The Netherlands, Germany, Slovakia, and Romania only adopted anti‐discrimination legislation in the labor market pursuant to the European Directive. This shows that, although a shift in focus was made in the 1990s and early 2000 to anti‐discrimination law on the international and EU level in line with the description of Waddington [1996], quota policies remain used in modern times. Furthermore, due to the coexistence of these policies and recent reinforcement of a quota system in the Netherlands, it is possible that anti‐discrimination policies did not achieve the increase in employment rates of people with disabilities, in contrast to the expectations of Sayce [2003]. Thus—based on the low employment rates of people with autism [Autism‐Europe, 2014]—investigating additional measures and support options for the promotion of employment for people with autism specifically on a policy level is recommended.

However, Waddington et al. [2016] explain how—regardless of the implementation of anti‐discrimination policy—little improvement is seen in the employment of people with disabilities and how the same applies to quota schemes. They suggest that a combination of policy initiatives may be key to addressing employment of disabilities. Their classification of policy initiatives consists of three categories: (a) the provision of public employment services; (b) employment training that helps people to increase their employment skills; and (c) subsidies to incentivize recruitment and work experience opportunities. When comparing these categories to the policies identified in the countries under study, it becomes apparent that very few of these categories are reflected in national policy. Instead, national policy is still very much concerned with a focus on human rights. As such, we can assert that—in terms of national policy—employment of people with disabilities is still in its infancy and requires more work, such as the creation of employment services and training for people with disabilities.

Findings of this study have to be seen in light of some limitations. First and foremost, a researcher bias may have played a role in the findings of this qualitative study. By solely focusing on quota and discrimination legislation, a confirmation bias might have affected the results. To reduce this bias, all reported findings were confirmed by an independent second author. A second limitation of the study was the electronically translated documents of documents in languages other than Dutch, English, and German, because of the risk of faulty interpretation. To account for this, speakers of all languages of countries under study, except, Slovakia and Romania verified the findings to the original documents. Similar mistakes could have occurred during data collection with the search strategy and title screening, although this part of the search was based on individual keywords or short key phrases, for which online translation services are suitable [Groves & Mundt, 2015]. Third, the legal databases were all accessed electronically and may not have contained all historic legal documents. To overcome this limitation the data collection contained a search for scientific articles as well, however, this does not fully guarantee completeness of the data. Fourth, there was a limited amount of contextual information found of Poland, Slovakia, and Romania in PubMed and Web of Science databases, in comparison to the other countries. This could have biased the results. Fifth, none of the countries had autism‐specific legislation. As such, we had to use general disability policy as a substitute for the analysis. While disability policy remains relevant to autism, it is less specific and can be considered less helpful than autism‐specific policies. Finally, only quota and anti‐discrimination legislation were addressed, disregarding other possibly relevant employment policies for persons with autism therefore limiting the implications of the research.

Despite an established framework of anti‐discrimination law in European and national employment policies since 2000, employment quotas are still in force in six out of the seven countries. This study shows the continuation of the shift in focus toward a social and anti‐discrimination approach of employment of people with disabilities, regardless of the presence of quota mechanisms. However, it is clear that neither of the policies have assured complete inclusion of people with autism in the European labor market. Thus, further research should focus on additional measures to increase the employment rate, such as employment programs that focus on counseling people with autism to integrate into the work floor and/or programs that educate the workforce on how to approach a co‐worker with autism [Waddington et al., 2016]. To accomplish this, the very first step that has to be taken is the collection of data on the employment rate of people with autism in each separate Member State to establish and exchange best‐practices, since this is still lacking. It is the duty of the EU and its Member States to address the unemployment problem of people with autism, in order to comply with the right of employment as established in the Universal Declaration of Human Rights, the Charter of Fundamental Rights of the EU, Directive 2000/78/EC, and Convention on the Rights of Persons with Disabilities, as well as many Constitutions and policies, to let people with autism be fully included in society.

## Conclusion

This study included employment policies from the United Nations, the EU and seven diverse EU Member States, with four different EU accession dates. The development of employment policies relating to people with autism appeared to be different for the Member States, though all shared the focus of adopting quota policies in the early stages of development. Critical junctures in the form of UN and EU policies gradually provided harmonization in a way that policy priorities were set on the topic of employment of people with disabilities. In six Member States, employment quotas are in force or reinforced, as well as extensive national anti‐discrimination frameworks in employment, which has its foundation in a European Directive. However, the employment rates of people with autism are still not equal to people without autism—indicating the need for further action.

## Conflict of Interest

There are no competing interests between the collaborating authors in this study.

## Author Contributions

Author A was in charge of writing the manuscript. Author B reviewed the manuscript after it was handed in as thesis and edited it to adhere to journal standards. All other authors reviewed the manuscript in its different stages and provided their input respectively, including contributions to the correct interpretation of the original language used in the legislation, suggesting additional documents for review missed during the initial search by the lead author, and suggesting revisions to the manuscript text.

## Data Availability

While all data are publicly available, a list of used documents along with their source has been included.

## Ethics Approval and Consent to Participate

Due to all data being publicly available and already in force in the respective Member States, the outcomes of this study have no ethical implications. Also, since the study was completely based off of public data, there was no situation in which it was necessary to request consent. Finally, neither sample sizes nor major demographic characteristics (aside from population size) were applicable to the study at hand. As such, these are not reported.

**Abbreviations**
EU: European Union
UK: United Kingdom
GDP: Gross Domestic Product
UN: United Nations

## Supporting information


**Appendix S1**: Supporting Information
