# Supplementary material for: Quotas, and Anti‐discrimination Policies Relating to Autism in the EU: Scoping Review and Policy Mapping in Germany, France, Netherlands, United Kingdom, Slovakia, Poland, and Romania
Source: Autism Res. 2020 May 22;13(8):1397–417. doi: 10.1002/aur.2315 (PMC7496597; doi:10.1002/aur.2315)
Supplement: Supplementary file 1 — Appendix S1: Supporting Information [file AUR-13-1397-s001.docx]

**United Nations**

United Nations. (1948). *Universal Declaration of Human Rights*. Retrieved from https://www.un.org/en/universal-declaration-human-rights.

United Nations. (1969). *Declaration on Social Progress and Development.* Retrieved from https://www.ohchr.org/Documents/ProfessionalInterest/progress.pdf.

United Nations. (1971). *Declaration on the Rights of Mentally Retarded Persons*. Retrieved from https://www.ohchr.org/EN/ProfessionalInterest/Pages/RightsOfMentallyRetardedPersons.aspx.

United Nations. (1975). *Declaration on the Rights of Disabled Persons*. Retrieved from https://www.ohchr.org/EN/ProfessionalInterest/Pages/RightsOfDisabledPersons.aspx.

United Nations. (1981). *The International Year of Disabled Persons.* Retrieved from https://www.un.org/development/desa/disabilities/the-international-year-of-disabled-persons-1981.html.

United Nations. (2006a). *Status of Treaties — Convention on the Rights of Persons with Disabilities*. Retrieved from https://treaties.un.org/Pages/ViewDetails.aspx?src=TREATY&mtdsg_no=IV-15&chapter=4&lang=_en&clang=_en.

United Nations. (2006b). *Convention on the Rights of Persons with Disabilities*. Retrieved from https://www.ohchr.org/EN/HRBodies/CRPD/Pages/ConventionRightsPersonsWithDisabilities.aspx.

**European Union**

Autism-Europe. (1992). *Charter for Persons with Autism* Retrieved from https://www.autismeurope.org/wp-content/uploads/2017/08/charter-for-persons-with-autism-1.pdf.

Council of the European Union. (1971). *Council Decision of 1 February 1971 on the reform of the European Social Fund*. Retrieved from https://eur-lex.europa.eu/legal-content/EN/TXT/PDF/?uri=CELEX:31971D0066&qid=1559037549923&from=EN.

Council of the European Union. (1986). *Council Recommendation of 24 July 1986 on the employment of disabled people in the Community*. Retrieved from https://eur-lex.europa.eu/legal-content/EN/TXT/?qid=1559037549923&uri=CELEX:31986H0379.

Council of the European Union. (2000). *Council Directive 2000/78/EC of 27 November 2000 establishing a general framework for equal treatment in employment and occupation*. Retrieved from https://eur-lex.europa.eu/legal-content/EN/TXT/?uri=celex%3A32000L0078.

European Commission. (1981). *The social integration of disabled people: A framework for the development of Community action*. Retrieved from https://eur-lex.europa.eu/legal-content/EN/TXT/?qid=1559037549923&uri=CELEX:31981Y1231(08).

European Commission. (1994). *White Paper on European Social Policy - A way forward for the Union*. Retrieved from https://eur-lex.europa.eu/legal-content/EN/ALL/?uri=COM:1994:0333:FIN.

European Parliament. (2015). *Written declaration, under Rule 123 of Parliament’s Rules of Procedure, on education and employment for persons with autism spectrum disorders and similar challenges*. Retrieved from http://www.europarl.europa.eu/sides/getDoc.do?pubRef=-//EP//NONSGML+WDECL+P7-DCL-2013-0025+0+DOC+PDF+V0//EN&language=EN

European Union. (1997). *Treaty of Amsterdam amending the Treaty on European Union, the Treaties establishing the European Communities and certain related acts*. Retrieved from https://eur-lex.europa.eu/legal-content/EN/TXT/?uri=CELEX%3A11997D%2FTXT.

European Union. (2000). *Charter of Fundamental Rights of the European Union*. Retrieved from https://www.europarl.europa.eu/charter/pdf/text_en.pdf.

European Union. (2010). *European Disability Strategy (2010-2020)*. Retrieved from https://eur-lex.europa.eu/legal-content/EN/TXT/?uri=LEGISSUM%3Aem0047.

**Germany**

Federal Republic of Germany. (1953). *Schwerbeschädigtengesetz [Severely Damaged Act]*. Retrieved from https://www.bgbl.de/xaver/bgbl/start.xav?start=%2F%2F*%5B%40attr_id%3D%27bgbl153s0389.pdf%27%5D#__bgbl__%2F%2F*%5B%40attr_id%3D%27bgbl153s0389.pdf%27%5D__1562261749995.

Federal Republic of Germany. (1974). *Schwerbehindertengesetz [Severely Handicapped Persons Act]*. Retrieved from https://www.bgbl.de/xaver/bgbl/start.xav?start=//*%5B@attr_id=%27bgbl174046.pdf%27%5D.

Federal Republic of Germany. (1994). *Gesetz zur Änderung des Grundsetzes vom 27. okotber 1994 [Act for Amendment of the Constitution of 27 October 1994]*. Retrieved from https://www.bgbl.de/xaver/bgbl/start.xav#__bgbl__%2F%2F*%5B%40attr_id%3D%27bgbl194s3146.pdf%27%5D__1561624788977.

Federal Republic of Germany. (2001). *Neuntes Buch Sozialgesetzbuch [Book Nine of the Social Code]*. Retrieved from http://www.gesetze-im-internet.de/sgb_9_2018/index.html#BJNR323410016BJNE005500000.

Federal Republic of Germany. (2002). *Behindertengleichstellungsgesetz [Disability Equality Act]*. Retrieved from https://www.gesetze-im-internet.de/bgg/BGG.pdf.

Federal Republic of Germany. (2006). Allgemeines Gleichbehandlungsgesetz [General Equal Treatment Act]. Retrieved from https://www.gesetze-im-internet.de/agg/BJNR189710006.html

**France**

French Republic. (1957). *Loi n°57-1223 du 23 novembre 1957 sur le reclassement des travailleurs handicapes [Act for the Employment of Disabled People]*. Retrieved from https://www.legifrance.gouv.fr/affichTexte.do?cidTexte=JORFTEXT000000880746&categorieLien=id.

French Republic. (1958). *Constitution du 4 octobre 1958 [Constitution of 4 October 1958]*. Retrieved from https://www.legifrance.gouv.fr/affichTexte.do?cidTexte=LEGITEXT000006071194.

French Republic. (1973). *Code du travail [Labour Code]*. Retrieved from https://www.legifrance.gouv.fr/affichCodeArticle.do;jsessionid=47A744A704D24DAD517738DEE2881B38.tplgfr43s_2?idArticle=LEGIARTI000006648640&cidTexte=LEGITEXT000006072050&categorieLien=id&dateTexte=19871231.

French Republic. (1975). *Loi n° 75-534 du 30 juin 1975 d'orientation en faveur des personnes handicapées [The Disability Orientation Act of 30 June 1975]*. Journal Officiel de la République Française Retrieved from https://www.legifrance.gouv.fr/affichTexte.do?dateTexte=20190610&cidTexte=JORFTEXT000000333976&fastPos=226&fastReqId=906012647&oldAction=rechExpTexteJorf.

French Republic. (1987). *Loi n° 87-517 du 10 juillet 1987 en faveur de l'emploi des travailleurs handicapés [Act of 10 July 1987 for the Employment of Disabled Workers* Retrieved from https://www.legifrance.gouv.fr/affichTexte.do?cidTexte=JORFTEXT000000512481.

French Republic. (1990). *Loi n° 90-602 du 12 juillet 1990 relative à la protection des personnes contre les discriminations en raison de leur état de santé ou de leur handicap [Act of 12 July 1990 on the Protection of Persons against Discrimination on Ground of their State of Health or Disability]*. Retrieved from https://www.legifrance.gouv.fr/affichTexte.do?cidTexte=JORFTEXT000000350518&dateTexte=.

French Republic. (2005). *LOI n° 2005-102 du 11 février 2005 pour l'égalité des droits et des chances, la participation et la citoyenneté des personnes handicapée [Act of Equal Rights and Opportunities, Participation and Citizenship of Persons with Disabilities]*. Retrieved from https://www.legifrance.gouv.fr/affichTexte.do?cidTexte=JORFTEXT000000809647&categorieLien=id.

**Netherlands**

The Netherlands. (1947). *Wet Plaatsing van Minder-valide Arbeidskrachten [Act of Placement of Disabled Workers]*. Retrieved from https://repository.overheid.nl/frbr/sgd/19461947/0000076895/1/pdf/SGD_19461947_0001253.pdf.

The Netherlands. (1981). *Wet arbeid gehandicapte werknemers [Handicapped Workers Employment Act]*. (17 384 ). Retrieved from https://repository.overheid.nl/frbr/sgd/19811982/0000155235/1/pdf/SGD_19811982_0005830.pdf.

The Netherlands. (1983). *Grondwet [Constitution]*. Retrieved from https://wetten.overheid.nl/BWBR0001840/2017-11-17.

The Netherlands. (1986). *Wet arbeid gehandicapte werknemers [Handicapped Workers Employment Act]*. Retrieved from http://deeplinking.kluwer.nl/?param=0035274D&cpid=WKNL-LTR-Nav2.

The Netherlands. (2003). *Wet gelijke behandeling op grond van handicap of chronische ziekte [Equal Treatment Disability & Illness Act]*. Retrieved from https://wetten.overheid.nl/BWBR0014915/2017-01-01.

The Netherlands. (2014). *Wijziging van de Wet financiering sociale verzekeringen in verband met een heffing bij het niet voldoen aan de quotumdoelstelling (Amendment to the Social Insurance Financing Act in connection with a levy in the event of non-compliance with the quota objective]*. Retrieved from https://zoek.officielebekendmakingen.nl/kst-33981-3.

The Netherlands. (2015). *Wet banenafspraak en quotum arbeidsbeperkten. [Jobs and Jobs Quota (Work Disabled Persons) Act]*. Retrieved from https://wetten.overheid.nl/BWBR0036551/2015-05-01.

The Netherlands. (2016). *Wet van 14 april 2016 tot uitvoering van het op 13 december 2006 te New York tot stand gekomen Verdrag inzake de rechten van personen met een handicap [Act of 12 April 2016 on implementation of CRPD]*. Retrieved from https://zoek.officielebekendmakingen.nl/stb-2016-216.

The Netherlands. (2018). *Wet banenafspraak en quotum arbeidsbeperkten Kennisdocument (versie voorjaar 2018) [Factsheet Jobs and Jobs Quota (Work Disabled Persons) Act]*. Retrieved from https://www.rijksoverheid.nl/documenten/publicaties/2015/03/06/kennisdocument.

The Netherlands. (2019). *Wijziging van de Wet financiering sociale verzekeringen, de Ziektewet en de Wet tegemoetkomingen loondomein, teneinde het deactiveren van de quotumheffing mogelijk te maken en erin te voorzien dat de quotumheffing niet eerder dan over het jaar 2022 wordt geheven en enige andere wijzigingen [Amendmet Jobs and Jobs Quota (Work Disabled Persons) Act]*. Retrieved from https://www.eerstekamer.nl/behandeling/20190122/gewijzigd_voorstel_van_wet/document3/f=/vkvgk1kyn5wn_opgemaakt.pdf.

**United Kingdom**

United Kingdom of Great Britain and Northern Ireland. (1944). *Disabled Persons (Employment) Act* Retrieved from http://www.legislation.gov.uk/ukpga/Geo6/7-8/10/enacted.

United Kingdom of Great Britain and Northern Ireland. (1995). *Disability Discrimination Act 1995*. Retrieved from http://www.legislation.gov.uk/ukpga/1995/50/contents?text=Employment%20AND%20disability#match-1.

United Kingdom of Great Britain and Northern Ireland. (2003). *The Disability Discrimination Act 1995 (Amendment) Regulations 2003*. Retrieved from https://www.legislation.gov.uk/uksi/2003/1673/regulation/7/made.

United Kingdom of Great Britain and Northern Ireland. (2005). *Disability Discrimination Act*. Retrieved from http://www.legislation.gov.uk/ukpga/2005/13/enacted#text%253DEmployment%2520AND%2520disability.

United Kingdom of Great Britain and Northern Ireland. (2010). *Equality Act* Retrieved from http://www.legislation.gov.uk/ukpga/2010/15/enacted.

**Slovakia**

Slovak Republic. (1992). *Ústava Slovenskej republiky [Constitution of the Slovak Republic]*. Retrieved from https://www.slov-lex.sk/pravne-predpisy/SK/ZZ/1992/460/20190701.

Slovak Republic. (1996). *Zákon Národnej rady Slovenskej republiky o zamestnanosti [Act on Employment]*. Retrieved from https://www.slov-lex.sk/pravne-predpisy/SK/ZZ/1996/387/20040101.html#predpis.cast-desiata.

Slovak Republic. (2001). *Zakonnik Práce z 2. júla 2001 [Labour Code of 2 July 2001]*. Retrieved from https://www.slov-lex.sk/pravne-predpisy/SK/ZZ/2001/311/vyhlasene_znenie.html.

Slovak Republic. (2004a). *o rovnakom zaobchádzaní v niektorých oblastiach a o ochrane pred diskrimináciou a o zmene a doplnení niektorých zákonov (antidiskriminačný zákon) [Anti-discrimination Act]*. Retrieved from https://www.slov-lex.sk/pravne-predpisy/SK/ZZ/2004/365/vyhlasene_znenie.html.

Slovak Republic. (2004b). *Zákon o službách zamestnanosti a o zmene a doplnení niektorých zákonov [Act on Employment Services and on Amendments and Supplements to certain Acts]*. Retrieved from https://www.slov-lex.sk/pravne-predpisy/SK/ZZ/2004/5/20190101.html#prilohy.

**Poland**

Republic of Poland. (1952). *Konstytucja Polskiej Rzeczypospolitej Ludowej 1952 [The Constitution of the Polish People's Republic 1952]*. Retrieved from http://dziennikustaw.gov.pl/DU/1952/s/33/232/1.

Republic of Poland. (1967). *Rozporządzenie Rady Ministrów z dnia 5 maja 1967 r. w sprawie planowego zatrudniania inwalidów [Ordinance of the Council of Ministers of 5 May 1967 on the Planned Employment of Disabled Persons]*. Retrieved from http://prawo.sejm.gov.pl/isap.nsf/DocDetails.xsp?id=WDU19670200088.

Republic of Poland. (1991). *Ustawa z dnia 9 maja 1991 r. o zatrudnianiu i rehabilitacji zawodowej osób niepełnosprawnych [Act of 9 May 1991 on Employment and Vocational Rehabilitation of the Disabled]*. Retrieved from http://prawo.sejm.gov.pl/isap.nsf/DocDetails.xsp?id=WDU19910460201.

Republic of Poland. (1996). *Ustawa z dnia 2 lutego 1996 r. o zmianie ustawy - Kodeks pracy oraz o zmianie niektÛrych ustaw [Act of February 2 1996 amending the Act - Labor Code and amending certain acts.]*. Retrieved from http://prawo.sejm.gov.pl/isap.nsf/DocDetails.xsp?id=WDU19960240110.

Republic of Poland. (1997a). *Konstytucja Rzecypopolitej Polskiej [Constitution of the Republic of Poland]*. Retrieved from https://www.prawo.vulcan.edu.pl/przegdok.asp?qdatprz=akt&qplikid=548.

Republic of Poland. (1997b). *Uchwała Sejmu Rzeczypospolitej Polskiej z dnia 1 sierpnia 1997 r. - Karta Praw Osób Niepełnosprawnych. [Charter of the Rights of Persons with Disabilities]*. Retrieved from http://prawo.sejm.gov.pl/isap.nsf/DocDetails.xsp?id=WMP19970500475.

Republic of Poland. (1997c). *ustawa z dnia 27 sierpnia 1997 r. o rehabilitacji zawodowej i społecznej oraz zatrudnianiu osób niepełnosprawnych [Act of 27 August 1997 on vocational and social rehabilitation and employment of disabled persons]*. Retrieved from http://prawo.sejm.gov.pl/isap.nsf/DocDetails.xsp?id=WDU19971230776.

Republic of Poland. (2004). *Ustawa z dnia 20 kwietnia 2004 r. o zmianie i uchyleniu niektórych ustaw w związku z uzyskaniem przez Rzeczpospolitą Polską członkostwa w Unii Europejskiej [Act of 20 April 2004 on the amendment and repeal of certain acts in connection with the Republic of Poland's accession to the European Union]*. Retrieved from http://prawo.sejm.gov.pl/isap.nsf/DocDetails.xsp?id=WDU20040960959.

Republic of Poland. (2010). *Ustawa z dnia 3 grudnia 2010 r. o wdrożeniu niektórych przepisów Unii Europejskiej w zakresie równego traktowania [Act on the implementation of certain European Union Regulations in the Field of Equal Treatment]*. Retrieved from http://prawo.sejm.gov.pl/isap.nsf/DocDetails.xsp?id=WDU20102541700.

**Romania**

Romania. (1964). *Constituţia din 10 iulie 1964 [Constitution of 10 July 1964]*. Retrieved from http://legislatie.just.ro/Public/DetaliiDocument/70595.

Romania. (1971). *Lege nr. 12 din 21 octombrie 1971 privind încadrarea şi promovarea în munca a personalului din unităţile socialiste [Act of 21 October 1971 on Employment and Promotion of Personnel from State Socialist Units]*. Retrieved from http://legislatie.just.ro/Public/DetaliiDocument/264.

Romania. (1976). *Lege nr. 25 din 5 noiembrie 1976 privind încadrarea într-o munca utila a persoanelor apte de muncă [Law no. 25 of November 5 1976 concerning the employment of workers capable of working]*. Retrieved from http://legislatie.just.ro/Public/DetaliiDocument/20009.

Romania. (1991). *Constituție din 21 noiembrie 1991 [Constitution of 21 November 1991]*. Retrieved from http://legislatie.just.ro/Public/DetaliiDocument/1413.

Romania. (1999). *Ordonanţa de Urgenţă nr. 102 din 29 iunie 1999 privind protecţia specială şi încadrarea în munca a persoanelor cu handicap [Emergency Ordinance of on the Special Protection and Employment of Persons with Disabilities]*. Retrieved from http://legislatie.just.ro/Public/DetaliiDocumentAfis/18606.

Romania. (2003a). *Codul Muncii [Labour Code]*. Retrieved from http://legislatie.just.ro/Public/DetaliiDocument/41627.

Romania. (2003b). *Lege nr. 429 din 23 octombrie 2003 de revizuire a Constituţiei României [Act of 23 October 2003 on the revision of the Constitution of Romania]*. Retrieved from http://legislatie.just.ro/Public/DetaliiDocumentAfis/47259.

Romania. (2004). *LEGE nr. 343 din 12 iulie 2004 pentru modificarea şi completarea Ordonanţei de urgenţă a Guvernului nr. 102/1999 privind protecţia specială şi încadrarea în muncă a persoanelor cu handicap [Act of 12 July 2004 amending and supplementing the Government Emergency Ordinance no. 102/1999 on the special protection and employment of persons with disabilities]*. Retrieved from http://legislatie.just.ro/Public/DetaliiDocument/53538.

Romania. (2006). *Lege nr. 448 din 6 decembrie 2006 privind protecția și promovarea drepturilor persoanelor cu handicap [Act on the Protection and Promotion of the Rights of Persons with Disabilities]*. Retrieved from http://legislatie.just.ro/Public/DetaliiDocumentAfis/88315.

Romania. (2017). *Ordonanta de Urgenta nr. 60 din 4 august 2017 pentru modificarea și completarea Legii nr. 448/2006 privind protecția și promovarea drepturilor persoanelor cu handicap [Emergency Ordinance of 4 August 2017 for amending and completing the Law no. 448/2006 on the protection and promotion of the rights of persons with disabilities]*. Retrieved from http://legislatie.just.ro/Public/DetaliiDocumentAfis/192155.
